# Impact of the implementation of an embedded palliative care model in the continuum of care for patients with metastatic breast cancer

**DOI:** 10.1007/s00520-025-09502-w

**Published:** 2025-05-05

**Authors:** Leonor Vasconcelos de Matos, Tatiana Louro, Teresa Gantes Padrao, Marcio Debiasi, Berta Sousa, Helena Gouveia, Fatima Cardoso

**Affiliations:** Breast Unit, Champalimaud Clinical Center, Champalimaud Foundation, Avenida de Brasília, S/N, 1400-038 Lisbon, Portugal

**Keywords:** Breast cancer, Metastatic, Palliative care

## Abstract

**Purpose:**

Timely and integrated palliative care is crucial for patients with metastatic breast cancer. However, data on models of integration are scarce. We aimed to evaluate the impact of the integration of an embedded model of palliative care in a multidisciplinary breast unit on important goals of care and to characterize different patterns of integration (palliative predominant, oncology predominant or concurrent).

**Methods:**

Single-center, retrospective, observational cohort study including all patients with metastatic breast cancer followed by the palliative and oncology teams from a 12-month period before (pre-implementation) and after (post-implementation) of an embedded model of integration of palliative care. We analyzed early integration, 1-year survival rate, survival and different patterns of coordination of palliative care and oncology (the oncology-predominant pattern, the palliative care-predominant pattern and the concurrent integrated care pattern).

**Results:**

From April 2020 to April 2022, a total of 145 patients were included in the analysis: all female, median age of 63.5 years, 20.7% with triple negative disease. Post-implementation, early referrals significantly increased (35.3 to 61.3%, *p* < 0.01), 1-year survival rate (40.1% vs 40.7%) and survival time were similar (9.2 months vs 9.9 months). An integrated pattern of care with concurrent palliative and oncology appointments was significantly more frequent (30% vs 61%, *p* < 0.01). When compared to the other patterns, the concurrent pattern was associated to a median of 4 months longer survival (*p* < 0.01).

**Conclusions:**

The incorporation of an embedded model of palliative care was associated with earlier referrals and translated into better outcomes for patients with metastatic breast cancer.

## Introduction

Approximately 5–7% of breast cancer (BC) is diagnosed as de novo metastatic, and up to one third of early BC will develop systemic recurrence [1]. However, breakthrough advances in the care of metastatic breast cancer (MBC) in the last years are changing mortality rates, and increments in survival of women with metastatic disease are slowly but steadily surfacing [2].

With more years to live with MBC, attention to the quality of these years is paramount. In the setting of incurable and advanced cancer, palliative care (PC) arises as an indispensable component of patient-centered care. In 2016, the American Society of Clinical Oncology (ASCO) recommended the need for integrative palliative care early in the disease-trajectory, alongside cancer-directed treatments [3].

Evidence shows how early integration of PC in the continuum of cancer care, predominantly through outpatient clinics, has immediate as well as downstream effects, as improved quality of life, better symptom management, more effective communication and attainment of goals, improved satisfaction of patients and caregivers, leading also to prolonged survival [4–7]. At a broader level, benefits extend to health care systems, with reduced health care costs resulting from less admissions to the emergency room and fewer hospitalizations, with more people dying at home [8–10]. Integration of PC and oncology can maximize the strengths of both specialties, as importantly stated in the European Society of Medical Oncology (ESMO) position paper on supportive and palliative care [11]. High quality data has been published showing that the integration of early PC interventions in the cancer care trajectory improve end-of-life care [5, 6, 9], but few details are available regarding these integrative pathways [12] and data come especially from mixed cancer samples which include a minority of patients with BC [13, 14].

Clinical models of integration of PC and oncology are often based on location of care, and within the outpatient hospital setting these include independent clinics and embedded models [15–17]. Regarding these last ones, which operate out of a shared space within the oncology clinic, the geographical proximity promotes communication, collaboration and coordination of supportive and PC, elevates discussion of patients’ cases and speeds the provision of palliative care, while providing a smoother referral [16]. However, and despite the great advantages of this model of care, data are lacking regarding experience with embedded models in the clinical setting, particularly concerning coordination and joint work amid the PC and oncology teams.

This study aimed to determine whether the introduction of an embedded model of PC for patients with MBC followed at a specialized Breast Care Unit affected outcomes of end-of-life care, such as the place of death, chemotherapy in the last 30 days of life or admission in emergency department services[18, 19], and survival. We also intended to study the role of early integration of PC into the continuum of the disease and to analyze and characterize the different patterns of integration of PC in the patient’s disease trajectory and their differential impact on survival.

## Methodology

### Definition of the setting

The European Society of Breast Cancer Specialists (EUSOMA) certified Breast Unit of the Champalimaud Clinical Center has adopted since April 2021 an embedded model of PC integration in the care of patients with MBC. The embedded clinic comprises one PC specialist, working full time and actively participating in the multidisciplinary discussions, backed by a team of specialized nurses with direct coordination with an inpatient acute PC unit and a home-visiting team, as well as other important allied health professionals available as needed, including psycho-oncology. This team and the oncology team work in close collaboration and partnership and together contribute to the provision of multidisciplinary and patient-centered care. This model offers scheduled appointments as well as urgent same-day consultations for patients with uncontrolled symptoms or other urgent needs. A nurse-led telephone triage line is available every day 12 h a day, for patients at home who experience acute uncontrolled symptoms. Additionally, and since the COVID-19 pandemic, teleconsultations have become a frequently and accepted method to follow patients [16, 20].

Before this model was introduced, PC was delivered as demanded by physicians and upon request, without a structured team to deliver this care. With the implementation of an embedded organization of PC within our Breast Unit, coordination and communication became facilitated. However, referral remained oncologist-initiated and guided by non-strict referral criteria as follows: moderate to severe physical or emotional symptoms, assistance with advance care planning and progressive disease after several lines of therapy.

### Study design

We have designed a single center, retrospective, observational cohort study that included all consecutive patients with a diagnosis of MBC that were followed by the PC team from April 2020 until April 2022. A 12-month period before (pre-implementation) and after (post-implementation) the introduction of an embedded PC model was considered the adequate time to provide a reliable longitudinal assessment of the effect of the introduction of a specialized PC model. Patients were included if they had histological confirmed diagnosis of breast cancer and confirmed metastatic disease (stage IV, according to the American Joint Committee on Cancer 8 th Edition) and had at least three appointments with the PC team of the Breast Unit, considered by the research team as the minimum visits to allow to measure the impact of PC interventions. Data were retrieved from electronic patient files and included the collection of data on baseline demography characteristics; breast cancer biological and clinical features; breast cancer treatments; survival status and place of death. Data on incidence and prevalence of MBC were gathered from Breast Care, an institutional structured database with ongoing collection of prespecified data from the medical records of patients treated at the Breast Unit.

The first PC appointment was considered the point of PC integration. The evaluated outcomes were “early integration” of PC, defined as: (1) first PC visit happening less than 3 months since the diagnosis of triple negative MBC or (2) first PC visit happening before third line of anticancer treatment, if non-triple negative disease; 1-year survival, calculated based on vital status from enrollment with the PC team to 1 year after enrollment and survival time, defined as the number of months from the date of enrollment with the PC team until death. Data from patients who were alive at the last available follow-up were censored on that date.

In the overall population, we identified three patterns of integration of PC and oncology: the oncology-predominant pattern (ONC), in which patients would have mainly oncology appointments (> 50% of total medical appointments in the Breast Unit) since the start of integration within the PC team; the PC-predominant pattern (PALL), in which patients were primarily followed by the PC team (> 50% of total medical appointments in the Breast Unit); and the concurrent integrated care pattern (CONC), characterized by a predominance of medical consultations that migrated between the PC specialist and the oncologist and were often performed with both specialists together (variation of number of PC and Oncology appointments < 20%).

This study was approved by the Champalimaud Foundation Institutional Review Board (IRB) on the 25 th July 2023, and all research procedures were conducted in accordance with the ethical standards outlined in the Declaration of Helsinki and relevant local regulations. Informed consent waiver request was approved by the IRB.

### Statistical analysis

A descriptive analysis was performed to characterize the study population, using frequencies and percentage for categorical variables and medians/range (min and max) for continuous variables. Comparisons between groups (pre versus post-implementation) were performed using the Mann–Whitney rank sum test for continuous variables and Chi square or Fisher’s exact test for categorical variables. A 1-year survival was chosen as specific outcome of study since the studied intervention most likely influences survival during the first year. Survival analysis was carried out and estimates performed using Kaplan–Meier method and between group differences for survival rates tested for significance using Cox’s proportional hazards models and the log-rank test. All the analysis were performed using STATA 15.1 software.

## Results

**Fig. 1:**
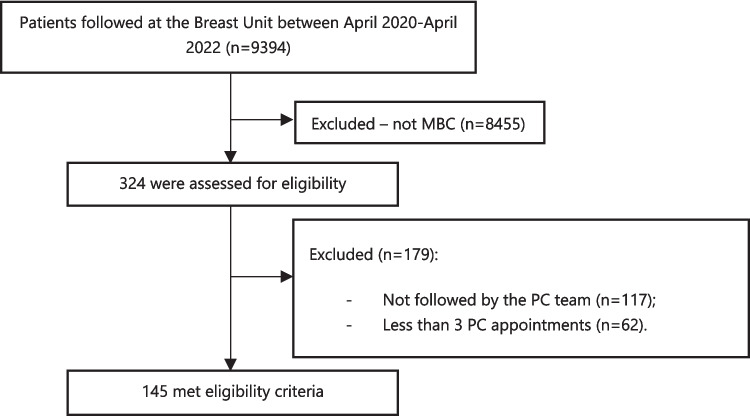
Patient enrollment

From April 2020 until April 2022, a total of 324 women were followed at the Breast Unit of the Champalimaud Clinical Center due to MBC (159 patients from April 2020 to March 2021 and 165 from April 2021 to April 2022). In this period, a total of 145 patients with MBC were followed by the PC team and met the inclusion criteria for the analysis (Fig. 1).

All patients were female, with a median age of 63.5 years (range 31–89). Most patients had a systemic recurrence (75%) of BC, while 25% had de novo MBC. Most patients had estrogen receptor positive (ER)/human epidermal growth factor receptor-2 (HER2) negative MBC (66.2%) and 20.7% had triple negative disease. Regarding location of metastasis at baseline, 46.8% patients had lung involvement, 55.8% liver metastasis and 17.2% presented with brain metastasis. Table 1 summarizes the demographic and clinical characteristics of the study population.

**Table 1:** Demographic and clinical patients’ characteristics

Variable	Overall population (*n* = 145)	ONC (*n* = 34)	PALL (*n* = 31)	CONC (*n* = 80)
Age				
Median (range)—years	63 (31–89)	61 (31–89)	63 (34–86)	64 (43–85)
Diagnosis of MBC, no (%)				
Metastatic disease at diagnosis	36 (25)	8 (23.5)	6 (19.3)	22 (27.5)
Systemic recurrence	109 (75)	26 (76.5)	25 (80.6)	58 (72.5)
Breast cancer biologic subtypes, no (%)				
HR +/HER2 −	96 (66.2)	21 (61.7)	19 (61.3)	56 (70)
HER2 +	19 (13.1)	9 (26.4)	2 (6.4)	8 (10)
HR −/HER2 −	30 (20.7)	4 (11.7)	10 (32.3)	16 (20)
Location of metastasis, no (%)				
Bone	128 (88.3)	33 (97.1)	26 (83.8)	69 (86)
CNS	25 (17.2)	4 (11.7)	9 (29.0)	12 (15)
Liver	81 (55.8)	20 (58.8)	15 (48.4)	46 (57.5)
Lung	67 (46.8)	14 (41.2)	13 (41.9)	40 (50)

*HR* hormone receptor, *HER2* human epidermal growth factor receptor 2, *CNS* central nervous system, *MBC* metastatic breast cancer

**Fig. 2:**
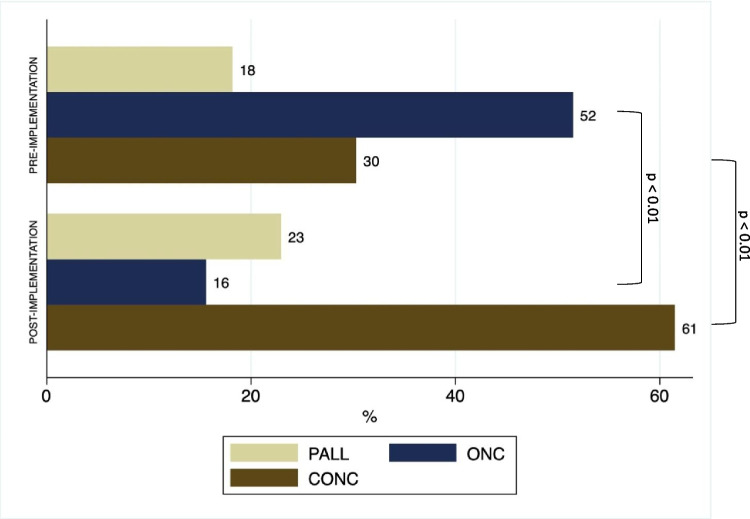
Patterns of PC and oncology integration, before and after the implementation of the embedded pc clinc

As can be observed in Fig. 2, the implementation of an embedded PC team was linked with an increase in PALL-led pattern (*p* < 0.01), a decrease in ONC-led pattern and a compensatory increase in an integrated concurrent pattern of oncology and PC visits (*p* < 0.01).

Patient characteristics in each pattern are presented in Table 1. Compared with other groups, in the PALL group there was a higher proportion of triple negative disease, and a higher proportion of central nervous system metastasis.

Table 2 summarizes data regarding patient care at and after admission to the PC consultations, before and after the implementation of an embedded PC clinic. In the pre-implementation period, patients followed by the PC team represented 21.4% (*n* = 34) of the total MBC population. In the post-implementation, this rate rose to 67.3% (*n* = 111). The introduction of an embedded integrated model was related with a statistically significant increase of 25.9% in early referrals (35.3 to 61.3%, *p* value = 0.008). Although without statistical significance, it was also associated to an increase of deaths at home or in PC units, and a decrease in emergency department and general hospital wards deaths.

**Table 2:** Patient care before and after implementation of embedded palliative care clinics

Variables	Overall population	Pre-implementation (*n* = 34)	Post-implementation (*n* = 111)	*p* value
Early integration of PC	80 (55.2)	12 (35.3)	68 (61.3)	** < 0.01**
No of lines of anticancer treatment before first PC appointment				
Median (range)	2 (0–11)	3 (1–11)	2 (0–9)	**0.01**
Place of death, no (%)				0.46
Home	29 (33)	6 (17.6)	23 (20.7)	
Palliative care unit	29 (33)	6 (17.6)	23 (20.7)	
Emergency department	2 (2)	1 (2.9)	1 (0.9)	
General hospital ward	29 (33)	10 (29.4)	19 (17.1)	
Last 30 days of life, no (%)				
Admitted in emergency department	24 (74)	3 (8.8)	21 (18.9)	0.64
Admitted in general hospital ward	23 (26)	6 (17.6)	17 (15.3)	0.11
Anticancer treatment	19 (23)	7 (20.6)	12 (10.8)	0.31
Main reason for referral to the palliative care team				0.45
Symptoms	108 (74.5)	30 (88)	78 (70.2)	
Several lines of anticancer treatment	24 (16.5)	3 (8.8)	21 (18.9)	
Diagnosis of metastatic breast cancer	12 (8.3)	1 (2.9)	11 (9.9)	

*PC* palliative care

**Fig. 3:**
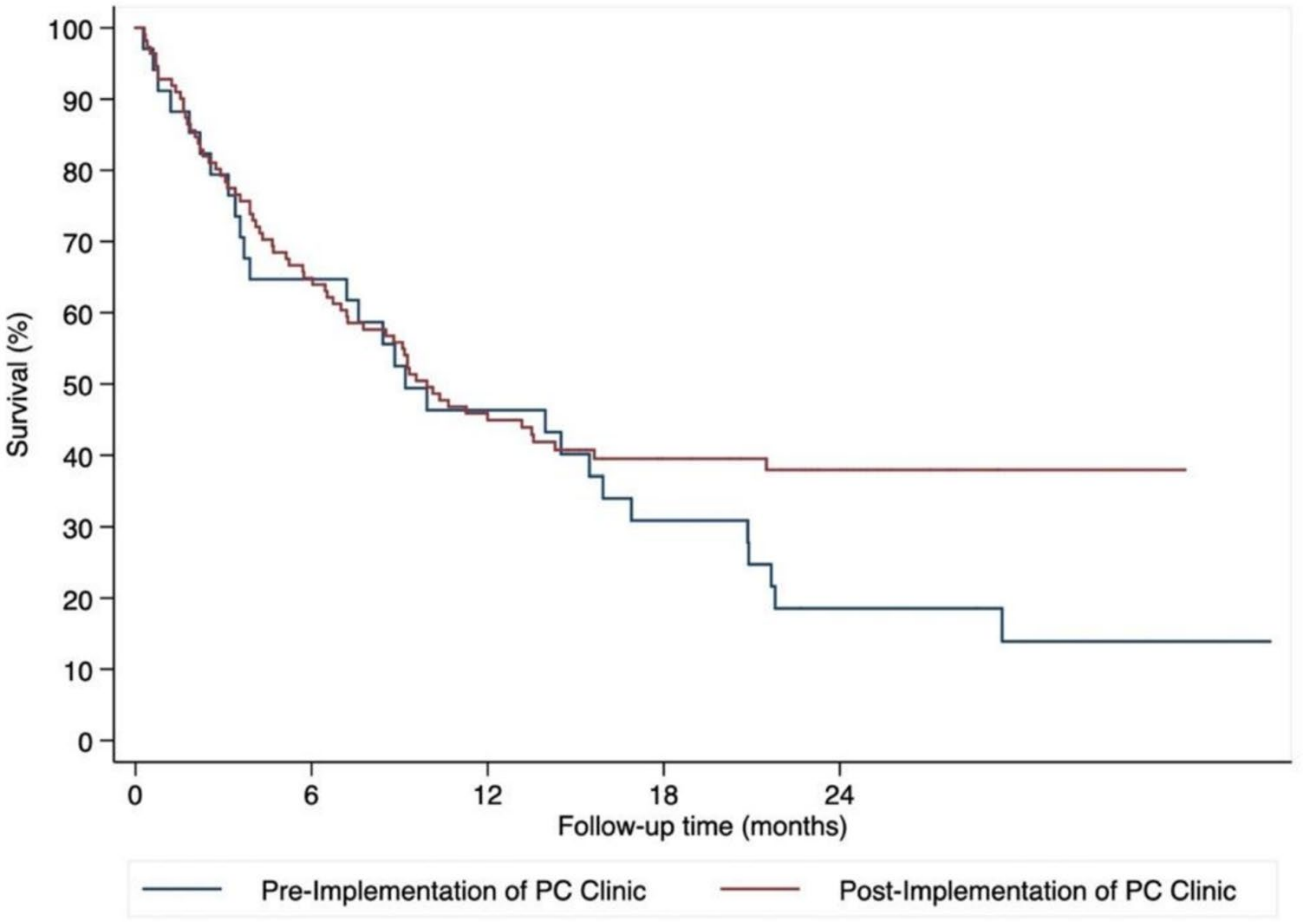
Kaplan–Meier curves for survival time in the analysis population, which included 145 patients, by study group

After a median follow-up of 23.3 months (range 3.5–20.2 months), the percentage of deaths was higher in the pre-implementation vs. post-implementation of the PC embedded clinic (87.5% vs 63.5% deaths, *p* = 0.010), although survival rate at 1-year was similar (40.1% pre-implementation, 95% CI 23.7–56.1 vs 40.7% post-implementation, 95% CI 31.4–49.8). Median survival time was also similar post-implementation (9.9 months vs 9.2 months, hazard ratio for death 0.74, 95% CI, 0.47–1.16; two-sided *p* = 0.19) (Fig. 3).

**Fig. 4:**
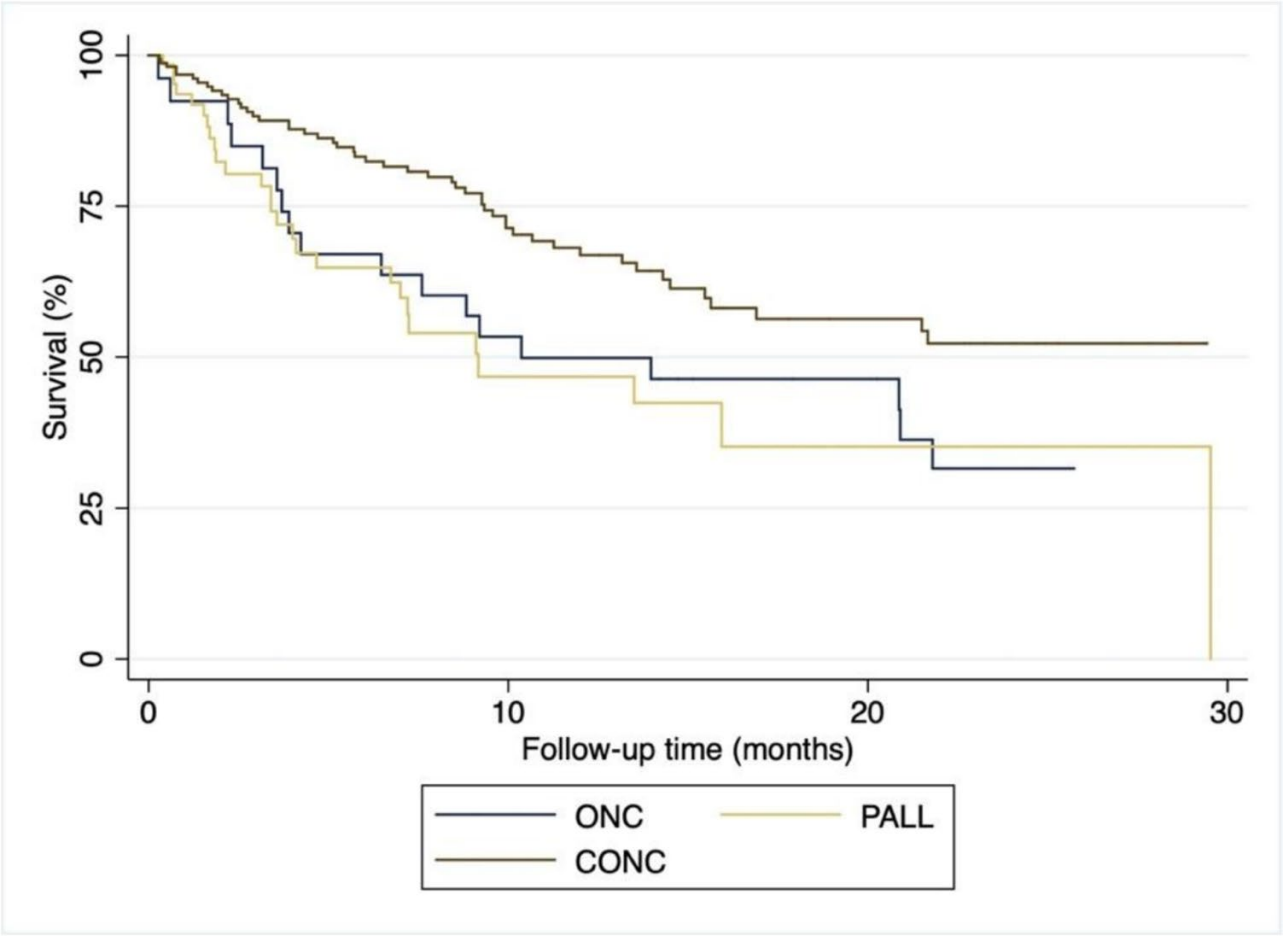
Kaplan–Meier curves for survival time in the analysis population, which included 145 patients, by model of integration of PC and Oncology. ONC oncology-led pattern, PALL palliative-led pattern, CONC concurrent integrated model of palliative care and oncology

Adjusting to age, location of metastasis and tumor biology, a concurrent model of integration was associated with a median of 4 months longer survival (7.6 months vs 11.3 months, hazard ratio for death 0.78; 95% CI 0.48–0.95, *p* < 0.01) (Fig. 4).

## Discussion

Our study allowed us to evaluate the impact of the integration of an embedded model of PC in the quality of end-of-life care and on survival of patients living with MBC. Indeed, this model allowed for a significant rise in a concurrent integrated pattern of palliative and oncology appointments, compensating a decrease in an oncology-led pattern that was more prevalent pre-implementation, which translated into prolonged survival. At the same time, earlier referrals to PC rose with the initiation of an embedded model of integration of PC. Patients with triple negative disease were more prevalent in this cohort of PC and oncology care, than in a general population of MBC, probably related to disease burden and symptom severity as well as worse prognosis, that triggered PC referral. Accordingly, there was a high prevalence of patients with visceral metastasis and CNS metastasis.

Unlike the disease courses of several metastatic solid tumors, patients with MBC often experience long disease trajectories. Also, the heterogeneity of disease subtypes is reflected on considerable variability in terms of disease prognosis. Thus, MBC provides perhaps the most challenging but also crucial field for establishing standardized criteria for PC integration, in order to ensure that all patients receive personalized and timely referral [21].

Collins et al. published the results of a population cohort study identifying, in MBC, multiday admission and presence of visceral metastasis as key transition points for PC integration [22]. Other retrospective analysis thus demonstrates that late referral of patients with MBC to PC is the norm and associated with shorter survival [23], while a randomized controlled trial conducted at Dana Farber showed how a structured PC intervention increases quality of end-of-life care for these patients(24). Additionally, some reports exist on inequalities of delivery of PC, especially end-of-life care, according to tumor biology [25]. A simultaneous care approach is suggested as an indicator of integration of oncology and PC, as well as care pathways and combined tumor boards [26]. Standardized care pathways have been proposed as a method to plan and implement complex clinical care like the integration of PC into cancer care and can be used to structure this approach [27].

This study evaluated the immediate 12 months before and after the implementation of an embedded model of integration of PC within a Breast Cancer dedicated Unit. This adaptation comprised several organizational constraints and adaptation to the model, and our results show the immediate downstream effects of this care shift that were, nevertheless, positive. Currently, this implementation is completely finalized and thus much smoother, which leads the authors to hypothesize that longer follow-up will probably lead to better outcomes.

Our study has some important limitations, which include its retrospective design and the fact that it is a single-center study. Also, additional relevant comorbidities, performance status and medication (such as nutritional supplements, corticosteroids, antidepressants, antipsychotics and antidiabetics) were not included in the analysis, and the authors recognize that some could affect the study outcomes. Additionally, the sample size may have limited the study power to detect significant effects of some variables, and thus type II error may affect the results. It is also important to state that, as a referral cancer center, occasionally patients only seek our Unit’s care in the setting of very advanced staged disease, which may confound our results especially in what concerns to timely referrals and end-of-life care.

We further recognize that our approach can be improved. The effect of PC involvement throughout the cancer journey can be further highlighted through the results obtained by the systematic collection of patient-reported outcomes [28]. Assessment of symptoms and quality of life is currently not just an important outcome of benefit in clinical trials; it is deemed to be performed in the clinical routine setting, refining the way clinicians evaluate treatments’ benefit, measure symptom severity and assess patient’s needs [29]. Furthermore, our embedded clinics worked with solely one PC specialist, when a minimum of two dedicated physicians would be needed to assure continued and transversal care, which should be considered when reflecting on the appropriate requirements of human resources.

Finally, it is unclear if embedded clinics are superior to stand alone PC clinics in regard to the frequency and timing of referrals and quality of care outcomes.

## Conclusion

In the past decades, deeper understanding of the molecular pathways and biologic subtypes of MBC has translated into improvements in cancer treatments and outcomes for patients with advanced disease. This understanding has been paralleled by increased complexity in symptom and side effects management, more demanding management of patients and family’s expectations as well as the increasing awareness on the importance to safeguard of quality of life.

Despite the strong evidence and robust endorsement by ASCO [30] and the Commission on Cancer [27], models for integration of outpatient PC and examples of successful implementation are still lacking. We believe that our work contributes to the importance of sharing real world examples of implementation, its strengths and weaknesses, in the hope to boost reproducibility in similar centers as well as to reflect on how to improve the care we are delivering to our patients.

We aim to reassess our practice with a longer timeframe, with an implemented framework of care and with improved resources. Additionally, a more contemporary analysis that reflects standard of care will also overtake some of the study’s limitations.

More awareness of this subject is needed, which can be enhanced not only through research, but especially through education. Emphasis on more training in PC during oncology residency curriculum can be an effective way, and increasing continued medical education on this topic is also very important for updated knowledge of PC and reduction of the stigma associated with end of life [31]. Effective communication is an essential component of integration [32].

The issue is not new, but the call for early and broader initiation of PC for patients with MBC is ever more compelling. The prevailing paradigms of a tumor-directed approach and a host-directed approach, as emphasized by the Oncology Commission [27], need to be merged together in order to thrive for the just cause. The required culture changes demand for collaboration between different stakeholders, including health care professionals, institutions, policymakers, funders and educators. Important recommendations have been issued, but they need to be put into practice in order to achieve the required paradigm shift in cancer care. We owe it to our patients and their families.

## Data Availability

No datasets were generated or analysed during the current study.
